# Dyspnea perception during the inspiratory resistive loads test in obese subjects waiting bariatric surgery

**DOI:** 10.1038/s41598-020-64677-y

**Published:** 2020-05-15

**Authors:** Karina Tomasini, Bruna Ziegler, Paulo Roberto Stefani Sanches, Danton Pereira da Silva Junior, Paulo Ricardo Thomé, Paulo de Tarso Roth Dalcin

**Affiliations:** 1 MSci, Nurse, Programa de Pós-Graduação em Ciências Pneumológicas, Universidade Federal do Rio Grande do Sul (UFRGS), Porto Alegre, Brazil; 2 Physiotherapist, Serviço de Fisioterapia, Hospital de Clínicas de Porto Alegre (HCPA); Programa de Pós-Graduação em Ciências Pneumológicas, UFRGS, Porto Alegre, Brazil; 3 Electrical engineer, Serviço de Pesquisa e Desenvolvimento em Engenharia Biomédica, HCPA, Porto Alegre, Brazil; 4 MD, Serviço de Pneumologia, HCPA; Professor of Medicine, Programa de Pós-Graduação em Ciências Pneumológicas, UFRGS, Porto Alegre, Brazil

**Keywords:** Diseases, Medical research

## Abstract

Identification of low dyspnea perception is relevant, since this condition is significantly associated with worse outcomes. We investigated dyspnea perception during the inspiratory resistive loads test on obese subjects waiting bariatric surgery in comparison with normal subjects. Secondarily, we analysed the proportion of obese subjects with low, moderate and high dyspnea perception. This observational study included subjects with body mass index (BMI) ≥ 35 kg/m^2^, compared to healthy subjects with BMI ≥ 18 and <25 kg/m^2^. Subject underwent clinical evaluation, inspiratory test with progressive resistive loads and spirometry. We studied 23 obese subjects (mean BMI = 51.9 ± 9.3 kg/m^2^) and 25 normal subjects (mean BMI = 24.3 ± 2.3 kg/m^2^). With the increase magnitude of resistive loads there was a significant increase in dyspnea score (p < 0.001) and progressive increase of the generated inspiratory pressure (p < 0.001), but there was no difference between the groups in terms of dyspnea score (p = 0.191) and no interaction effect (p = 0.372). Among the obese subjects, 4 individuals were classified as low perception, 11 as moderate and 8 as high. In conclusion, the degree of dyspnea perception during the inspiratory progressive resistive loads test did not differ between obese and normal subjects. Among obese subjects, only 17% were classified as low dyspnea perception.

## Introduction

Dyspnea is defined as an uncomfortable perception of breathing, inappropriate to physiological circumstances^1^. It is a sensation of respiratory distress experienced by patients suffering from various diseases and by healthy individuals under extreme exercise conditions^2^. It is a complex symptom that signs that breathing homeostasis could be compromised, and may lead to an adaptive response, such as resting or seeking medical attention^3,4^.

In recent years, obesity has become a global public health issue and it has been estimated that 2.3 billion adults are overweight and over 700 million adults are obese^5,6^.

Obesity is related to several abnormalities of respiratory mechanics due to impairment of the thorax and diaphragm, what leads to changes in ventilation and impairment of gas diffusion. Respiratory functional repercussion is directly proportional to the degree of obesity^7^.

Obese individuals present more dyspnea than normal individuals, both at rest and in exercise, even in the absence of cardiopulmonary diseases^8–10^. Studies on the prevalence of dyspnea and on the pathophysiological mechanisms of this symptom in obese individuals are scarce^11^. The assessment of dyspnea using objective methods that allow the quantification of this symptom have gained an increasing importance in this area of research. The identification of individuals with low perception of dyspnea is relevant, since this condition is significantly associated with worse outcomes regarding morbidity and mortality^12,13^. The use of the inspiratory resistive load system during ventilation is a simple, safe and practical approach to identify subjects with low dyspnea perception^14,15^.

The objective of this study was to assess the level of perception of dyspnea using progressive inspiratory resistive loads in obese individuals who are candidates for bariatric surgery, in comparison to normal individuals. The secondary objective was to analyze the proportion of obese individuals with low, moderate and high perception of dyspnea.

## Methods

This was a observational cross-sectional study^16,17^. Over the course of one week, obese and normal subjects underwent clinical assessment, inspiratory resistive load test and spirometry. The study was approved by the Research Ethics Committee of the Hospital de Clínicas de Porto Alegre (HCPA) under protocol number 11 0148. All participants signed the Informed Consent Form (ICF). All methods were performed in accordance with the international and national standards for clinical study in human (Declaration of Helsinki and Brazilian Governmental regulation – Plataforma Brasil).

The study population consisted of obese patients, candidates for bariatric surgery, who were being assessed at the outpatient clinic of the Pneumology Service of the HCPA. The inclusion criteria were: body mass index (BMI) equal to or greater than 35 kg/m^2^, with age equal to or greater than 18-year-old. The exclusion criteria were: active smoking or cessation of smoking for less than 6 months; smoking index greater than 5 packs-year; presence of any form of chronic lung disease, such as chronic obstructive pulmonary disease, asthma, bronchiectasis, tuberculosis sequelae; presence of heart failure; infection by the human immunodeficiency virus and/or presence of the human acquired immunodeficiency syndrome; inability to complete the resistive load test; presence of any condition that would prevent the completion of the tests proposed by the research protocol; respiratory infection in the previous 30 days; and pregnancy.

Normal subjects were selected through the affixation of notices in HCPA and online ads. The inclusion criteria for these subjects were: BMI equal to or greater than 18 kg/m^2^ and less than 25 kg/m^2^; age equal to or greater than 18-year-old. The exclusion criteria were: active smoking or cessation of smoking for less than 6 months; smoking index greater than 5 packs-year; presence of any form of chronic lung disease, such as chronic obstructive pulmonary disease, asthma, bronchiectasis, tuberculosis sequelae; presence of heart failure; infection by the human immunodeficiency virus and/or presence of the human acquired immunodeficiency syndrome; inability to complete the resistive load test; presence of any condition that would prevent the completion of the tests of the research protocol; respiratory infection in the previous 30 days; and pregnancy.

The perception of dyspnea was assessed using a system of inspiratory resistive loads, which comprises a Hans-Rudolph unidirectional valve (Hans Rudolph, Shawnee, Kansas, USA) and a rebreathing circuit^18^. A disk with eight holes of different diameters produces increasing inspiratory resistive loads of 0.6; 7.0; 15; 25; 46.7; 67 and 78 cmH_2_O/L/s, assuming a constant flow of 300 mL/s. Hans-Rudolph’s unidirectional respiratory valve separates the inspiratory flow from the expiratory and applies resistances only to the inspiration phase. The system was interconnected to a computer, making it possible to record the respiratory pressure curve. The mean inspiratory pressure, inspiratory time and respiratory rate at each level of resistive load were obtained through the respiratory pressure curve^13^. On the computer screen, the modified Borg scale^19^ was presented to the subject, ranging from 0 (absence of dyspnea) to 10 (maximum severity of dyspnea). Initially, individuals received information about the test and became familiar with the system. The subjects sat in front of the system, placed a nasal clip and were instructed to ventilate normally through a mouthpiece connected to the system. The participant ventilated at each level of resistive load for 2 minutes. After, the subject scored the dyspnea perception using the modified Borg scale displayed on the computer screen. Participants were free to choose frequency, volume, and respiratory flow to keep their respiratory pattern as natural as possible. In all the tests, a 1-minute pause was taken between each resistive load to assure the patient’s comfort and time to swallow saliva.

Spirometry was performed at the Pulmonary Physiology of HCPA, with a computerized spirometer (MasterScreen, V 4.3, Jaeger, Wuerzburg, Germany). Three technically acceptable forced expiratory curves were obtained^20^. The curve with the highest value was recorded. Forced expiratory volume in the first second (FEV_1_), forced vital capacity (FVC) and FEV_1_/FVC ratio were recorded. The values were expressed in liters and in percentage of predicted for sex, age and height^21^.

The classification of nutritional status was based on BMI, calculated by weight (kg) divided by height (m) squared.

The assessment of dyspnea in relation to activities of daily living was scored using the modified scale of the British Council for Medical Research (mMRC)^22^.

Peripheral saturation of oxyhemoglobin (SpO_2_) was measured with the subject at rest using pulse oximeter (NPB-40; Nellcor Puritan Bennett, Pleasanton, California, USA). End-tidal carbon dioxide (EtCO_2_) was measured with the subject at rest, using the Nonin Resp Sense Capnograph, Plymouth, Minnesota (USA).

The data were processed and analyzed with the Statistical Package for the Social Sciences (SPSS), version 18.0. A descriptive analysis was performed for the baseline characteristics of the study population. Quantitative data were presented as mean ± standard deviation (SD) or as median (interquartile range). Qualitative data were expressed in n (% of all cases). The analysis of variables with repeated measures (dyspnea perception scores, inspiratory pressure, respiratory rate and heart rate) was performed using the general linear statistical model for repeated measures with estimated equations, considering the participants of the study (obese and normal) as “subjects”, the dyspnea score at each inspiratory pressure level as “moments” and the behavior of each variable (dyspnea perception scores, inspiratory pressures, respiratory rate and heart rate) as “interaction”, throughout the resistive loads. The obese and normal patients were stratified according to the level of perception of dyspnea, with the inspiratory resistive load rating of 78.0 cmH_2_O/L/s, so that each patient was classified into a group of *low perception* (Borg score <4 points), *moderate perception* (Borg score of 4 to 8 points) or *high perception* (Borg score> 8 points). A comparative analysis was performed between the groups of perception of dyspnea (low, moderate and high) for obese individuals. Categorical variables were analyzed using the chi-square test. The quantitative variables with normal distribution were analyzed using the analysis of variance to 1 factor and the Tukey test for multiple comparisons. A correlation analysis with Spearman test was performed between level of perception of dyspnea at inspiratory resistive load rating of 78.0 cmH2O/L/s and FVC % predicted and FEV_1_% predicted. All statistical tests were two-tailed, and the statistical significance level was set at 0.05.

## Results

Thirty obese patients were examined. Of those, 2 patients were excluded because they were not able to tolerate the dyspnea perception test to completion, 4 because they had asthma diagnosis and 1 for being an active smoker. Therefore, 23 individuals with obesity were included in the study.

Thirty individuals who volunteered for the control group were examined. Five volunteers were excluded: two were not able to tolerate the dyspnea perception test to completion, one had asthma, one was an active smoker and one presented abnormal values in spirometry. Thus, 25 normal volunteers completed the study.

Table 1 shows characteristics of the participants of this study. In the obese group, mean age was 43.7 ± 12.1 years-old and the mean BMI was 51.9 ± 9.3 kg/m^2^. When comparing the groups (obese and normal individuals), there was no significant difference for age (p = 0.274), sex (p = 0.556) and race (p = 0.487). The predicted values of FEV_1_% and FVC% were significantly lower in obese than in normal subjects (respectively, 90.0 ± 14.4 versus 106.5 ± 21.5%, p = 0.003, and 92.0 ± 14.5% vs. 110.6 ± 19.5%, p < 0.001). There was a significant difference between the groups for mMRC score (p < 0.001), so that 100% of the normal subjects had a score equal to 0, while 34.8% of the obese had a score equal to 0, and 65.2% had score of 1.

**Table 1:** Characteristic of participants.

Variables	Obese n = 23	Normal n = 25	*P*
Age (years), mean ± SD	43.7 ± 12.1	39.8 ± 12.2	0.274
Sex (male/female), n (%)	7/16	10/15	0.556
Race (white), n (%)	17(73.9)	21(84)	0.487
BMI (Kg/m²), mean ± SD	51.9 ± 9.3	24.3 ± 2.3	<0.001
FEV_1_ (L), mean ± SD	2.7 ± 0.6	3.3 ± 0.8	0.006
FEV_1_ (% predicted), mean ± SD	90.0 ± 14.4	106.5 ± 21.5	0.003
FVC (L), mean ± SD	3.4 ± 0.8	4.0 ± 0.9	0.005
FVC (% predicted), mean ± SD	92.0 ± 14.5	110.6 ± 19.5	<0.001
FEV_1_/FVC, mean ± SD	81.5 ± 4.8	80.6 ± 6.5	0.575
FEV_1_/FVC (% predicted), mean ± SD	97.8 ± 6.2	96.2 ± 6.5	0.396
EtCO_2_ (mmHg), mean ± SD	40.3 ± 9.6	39.0 ± 5.4	0.548
SpO_2_ at rest (%), mean ± SD	97.4 ± 1.4	98.1 ± 1.4	0.065
Dyspnea at rest (scale mMRC), n (%)			
Grade 0	8 (34.8)	25 (100)	<0.001
Grade 1	15 (65.2)	0 (0)	

n = number of cases, BMI = body mass index, SD = standard deviation, FEV_1 _= forced expiratory volume in one second, FVC = forced vital capacity, EtCO_2 _= level of carbon dioxide released at the end of expiration, SpO_2 _= peripheral oxygen saturation, mMRC = Modified Medical Research Council Dyspnea Scale.

Student’s t test for independent samples; chi-square test for categorical variables.

The analysis among the obese subjects, stratified according to the score of dyspnea is presented in Table 2. Four individuals were classified as low perception, 11 as moderate and 8 as high. The median Borg score was 3 in the low perception group, 6 in the moderate group, and 10 in the high group. There was an association between age and group of dyspnea perception (p = 0.008), and mean age in the low perception group (58.0 ± 6.8 years-old) was significantly higher than in the high perception group (36.5 ± 9.3 years-old) and did not differ from the moderate perception group (36.5 ± 9.3 years-old); the moderate perception group did not differ significantly from the high perception group. There was no significant association between BMI and dyspnea perception(p = 0.271).

**Table 2:** Analysis among the obese groups, stratified according to the level of perception of dyspnea, with the inspiratory resistive load rating of 78.0 cmH_2_O/L/s.

Variable	Low (n = 4)	Moderate (n = 11)	High (n = 8)	P
Age (years), mean ± SD	58.0 ± 6.8 ^A^	43.6 ± 11.2^AB^	36.5 ± 9.3^B^	0.008
Sex (male/female), n	2/2	3/8	2/6	0.642
Dyspnea – MMRC scale (pontos), median (IA)	3 (2)^A^	6 (4)^B^	10 (2)^C^	< 0.0001
BMI (Kg/m^2^), mean ± SD	46.4 ± 5.4	51.3 ± 9.9	55.6 ± 9.3	0.271
PEFR (% predicted), mean ± SD	86.7 ± 26.1	91.5 ± 17.9	91.4 ± 13.2	0.891
FEV_1_ (% predicted), mean ± SD	81.3 ± 22.1	92.4 ± 13.2	91.0 ± 11.1	0.425
FVC (% predicted), mean ± SD	79.3 ± 19.1	94.9 ± 11.23	94.4 ± 14.5	0.160
FEV_1_/FVC (% predicted), mean ± SD	99.1 ± 12.8	97.7 ± 5.0	97.1 ± 3.7	0.879
IP (cmH_2_O) with IRL of 0.6 cm H_2_O/L/s,, mean ± SD	2.9 ± 0.7	3.2 ± 1.0	3.1 ± 0.9	0.778
IP (cmH_2_O) with IRL of 7.0 cm H_2_O/L/s, mean ± SD	6.2 ± 3.8	5.8 ± 2.9	4.3 ± 1.2	0.392
IP (cmH_2_O) with IRL of 15.0 cm H_2_O/L/s, mean ± SD	14.2 ± 12.7	8.9 ± 4.9	5.7 ± 1.9	0.104
IP (cmH_2_O) with IRL of 25.0 cm H_2_O/L/s, mean ± SD	17.3 ± 15.1	6.3 ± 1.9	7.5 ± 3.6	0.135
IP (cmH_2_O) with IRL of 46.7 cm H_2_O/L/s, mean ± SD	18.6 ± 16.7	12.9 ± 5.8	10.6 ± 8.4	0.372
IP (cmH_2_O) with IRL of 67.0 cm H_2_O/L/s, mean ± SD	19.3 ± 15.9	17.5 ± 8.3	13.4 ± 7.6	0.536
IP (cmH_2_O) with IRL of 78.0 cmH_2_O/L/s, mean ± SD	18.8 ± 16.6	17.3 ± 7.4	15.1 ± 8.4	0.806

n = number of cases, SD = standard deviation, mMRC = Modified Medical Research Council Dyspnea Scale, BMI = body mass index, PEFR = peak expiratory flow rate, FEV_1 _= forced expiratory volume in one second, FVC = forced vital capacity, IP = inspiratory pressure, IRL = inspiratory resistive load.

One-way analysis of variance with Tukey post-hoc test or Kruskal-Wallis with post-hoc Z test for continuous variables. In the post-hoc test, the letters A and B indicate that the means or medians are significantly different: A differs from B, and AB does not differ from A or B. Pearson chi-square test for proportions.

**Figure 1:**
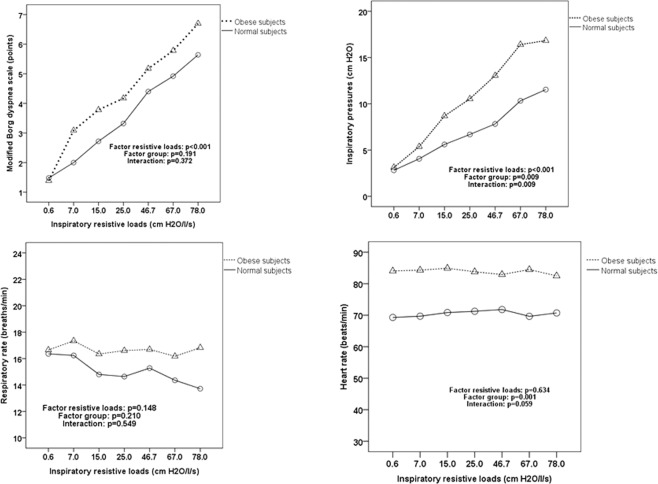
General linear analysis with repeated measures throughout the different resistive loads, stratified by group (obese versus normal individuals) with estimated equations for Borg dyspnea scale, inspiratory pressure, respiratory rate and heart rate.

Figure 1 shows general linear analysis with repeated measures throughout the different resistive loads, stratified by group (obese versus normal individuals) with estimated equations for dyspnea, inspiratory pressure, respiratory rate and heart rate scores. As resistive loads were increased, both dyspnea score (p < 0.001) and inspiratory pressure (p < 0.001) were progressively higher in obese and normal subjects, however there was no difference between the groups regarding dyspnea score (p = 0.191). There was no interaction effect (p = 0.372) between group and dyspnea score, that is, obese individuals and normal individuals presented similar behavior regarding dyspnea throughout the different resistive loads. Obese individuals generated higher inspiratory pressures than normal individuals (p = 0.009) and with progressive increase over different resistive loads (interaction effect = 0.009). Heart rate was significantly higher in obese subjects than in normal subjects (p = 0.001), however it did not change significantly over the resistive loads (p = 0.634) and there was no interaction effect (p = 0.059).

**Figure 2:**
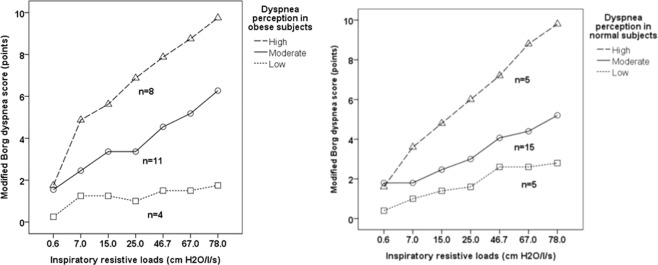
Obese and normal individuals stratified by level of dyspnea perception (tertiles of modified Borg dyspnea score), using inspiratory resistive loads of 78.0 cmH2O/l/s. Dyspnea perception groups (modified Borg dyspnea score): low (≤2.9); moderate (3–8.9) and high (≥9). Thera was no significant difference between obese and normal subjects for the proportion of low, moderate and high dyspnea score (p = 0.512).

Figure 2 shows the obese and normal individuals stratified by level of perception of dyspnea. There was no significant difference between obese and normal subjects for low, moderate and high dyspnea (p = 0.512).

Table 3 presents 95% confidence interval for dyspnea perception during the inspiratory resistive load testing.

**Table 3:** 95% confidence interval for dyspnea perception during the inspiratory resistive load testing.

Inspiratory resistive loads	Obese 95% CI	Normal 95% CI
0.6 cmH2O/L/s	0.79–2.17	0.65–2,14
7.0 cmH2O/L/s	1.36–2.64	1.97–4.21
15 cmH2O/L/s	1.92–3.52	2.66–4.90
25 cmH2O/L/s	2.51–4.13	2.99–5.36
46.7 cmH2O/L/s	3.44–5.36	3.89–6.46
67 cmH2O/L/s	3.83–6.01	4.39–7.17
78 cmH2O/L/s	4.58–6.70	5.38–8.01

CI = confidence interval.

The correlation analysis with Spearman test demonstrated no significant correlation for dyspnea score at inspiratory resistive load rating of 78.0 cmH2O/L/s and FVC % predicted (r = 0.037, p = 0.803) and FEV_1_% predicted (r = 0.106, p = 0.472).

## Discussion

This study shows that the level of perception of dyspnea during the test of progressive inspiratory resistive loads in obese patients did not differ from normal subjects. During ventilation in the system, obese individuals generated higher inspiratory pressures than normal individuals throughout increasing resistive loads, and those higher values were even greater in the resistive loads of >15 cmH_2_O/L/s. Among the obese subjects, 17% were classified as having low perception of dyspnea, 48% as moderate perception, and 35% as high perception. The proportion of individuals classified as low, moderate and high perception of dyspnea did not differ between obese and normal subjects. In the obese individuals, the low perception of dyspnea was associated with more advanced age. There was no association between level of perception of dyspnea and BMI.

A methodological aspect to be highlighted in this study is that all the tests were performed with a one-minute pause between each resistive load, allowing the patient comfort and saliva swallowing. This lowered the rate of withdrawal throughout the test, what was identified in other studies.

Few studies have sought to elucidate the epidemiology and pathophysiological mechanisms involved in dyspnea and obesity^11^. None of them used inspiratory resistive load test in the assessment. A Swedish study^23^ showed that 69% of 2,309 adults aged between 37–60 years-old with BMI ≥ 34 kg/m^2^ complained of dyspnea when they climbed two flights of stairs. A North American epidemiological study^10^ assessed 16,171 individuals aged ≥17 year-old and showed a positive association between BMI and prevalence of self-reported dyspnea on exercise.

Sahebjami^9^ investigated the presence of dyspnea at rest in obese individuals without other diseases. In a group of 23 men with BMI > 28 kg/m^2^, 15 reported dyspnea at rest and 8 denied having the symptom. Subjects with dyspnea had higher weight and higher BMI. Most individuals with dyspnea at rest were smokers or former smokers. In contrast, in the present study, active smokers were excluded and the criterion of <5 packs-year was used for former smokers.

Babb *et al*.^24^ assessed dyspnea on exercise in obese women with no other diseases. The authors demonstrated that dyspnea on exercise is prevalent in obese women and seems to be strongly associated with increased oxygen cost of breathing. There was no reduction in exercise capacity.

Bernhardt *et al*.^25^ studied 9 obese men who were assessed for body composition, fat distribution, lung function, cycle ergometry and oxygen cost of breathing analysis. Nine patients had dyspnea scores ≤ 2 on exercise and 10 had scores> 4. Among the obese, 37% had high dyspnea scores during exercise.

Ebihara *et al*.^12^ assessed the perception of dyspnea with inspiratory resistive loads of 10, 20 and 30 cmH_2_O/L/s in 479 elderly Japanese subjects with normal lung function and community residents. Patients were divided into tertiles according to the perception of dyspnea, which was classified as low in 153 individuals, intermediate in 160, and high in 166. The authors concluded that, among elderly residents of the community, the poor perception of dyspnea was related to hospitalization, high medical costs and all-cause mortality.

Ziegler *et al*.^13^ studied the dyspnea variability in 42 healthy subjects using an inspiratory resistive load system equal to the one used in this study (0.6, 7.0, 15, 25, 46.7, 67 and 78 cmH_2_O/L/s). Participants ventilated using the device for 2 minutes, but without pause between each resistive load. Scores of dyspnea presented wide variability. Perception of dyspnea was classified as low, intermediate and high in 31%, 45% and 24%, respectively. However, the cut-off points in the Borg scores used for this classification were different, making it impossible to compare them with the findings of the present study.

The present study has some limitations. First, the main limitation arises from the small sample size and, mainly, the inclusion of only obese individuals with BMI equal to or greater than 35 kg/m^2^, which prevents the generalization of the findings and limits the comparison of the clinical characteristics in the different groups of perception of dyspnea. The low number of participants is in no way a valid proof of the group’s comparability (absence of evidence is not evidence of absence)^26^ So, this study should be considered a descriptive exploratory research. Exploratory research intends merely to explore the research questions and does not intend to offer final and conclusive solutions to existing problems. Secondly, the cross-sectional design prevents the examination of temporal relations between perception of dyspnea and clinical outcomes. Thirdly, obese subjects waiting bariatric surgery represented a selected sample of the obese population that may have less comorbidities. Then the generalizability of the study is limited to this population.

In conclusion, the level of perception of dyspnea during the test of progressive inspiratory resistive loads in obese individuals who were candidates for bariatric surgery did not differ from normal individuals. Among the obese individuals, 17% were classified as low perception of dyspnea, 48% as moderate perception, and 35% as high perception. The proportion of individuals classified as low, moderate and high perception of dyspnea did not differ between obese and normal subjects. In obese individuals, low perception of dyspnea was associated with older age.

The practical implication of the present study is that obese subjects waiting bariatric surgery may have no additional risk for morbidity and mortality associated with low perception of dyspnea.

### Quick Look

This study shows that the level of dyspnea perception during the test of progressive inspiratory resistive in obese patients did not differ from normal subjects.

During ventilation in the system, obese individuals generated higher inspiratory pressures than normal individuals throughout increasing resistive loads.

The proportion of individuals classified as low, moderate and high perception of dyspnea did not differ between obese and normal subjects.

In the obese individuals, the low perception of dyspnea was associated with more advanced age.

There was no association between level of perception of dyspnea and BMI.
